# Comparison of Two Surgical Techniques for Implantation of the Ponto^®^ Bone Conduction Implant

**DOI:** 10.3390/jcm15166482

**Published:** 2026-08-21

**Authors:** Paulina Podlawska-Nowak, Wojciech Gawęcki, Anna Bartochowska

**Affiliations:** 1 Department of Otolaryngology, Regional Hospital named by Mikołaj Pirogov, 90-531 Łódź, Poland; paulinapodlawska@gmail.com; 2 Department of Otolaryngology and Laryngological Oncology, Poznan University of Medical Sciences, 60-355 Poznań, Poland; 3 Department of Phoniatrics and Audiology, Poznan University of Medical Sciences, 60-355 Poznań, Poland; annabartochowska@gmail.com

**Keywords:** bone-anchored hearing device, bone conduction implant, Ponto, Minimally Invasive Ponto Surgery (MIPS), linear incision technique, conductive hearing loss, single-sided deafness, adverse skin reaction

## Abstract

**Objectives**: The aim of this study was to compare the surgical and postoperative clinical outcomes of punch-only and linear incision techniques for implantation of the Ponto^®^ bone conduction implant. **Methods**: Surgical and follow-up data were collected from 101 patients: 46 treated with Minimally Invasive Ponto^®^ Surgery (MIPS) and 55 with the linear incision technique (LT). The analysis focused on: (1) the surgical procedure, including operative time and intraoperative complications; (2) the healing process; and (3) long-term implant-site outcomes, including implant loss. **Results**: Median operative time was significantly shorter for MIPS than for LT (30 min vs. 40 min, *p* < 0.001). No major intraoperative complications were observed in either group. Adverse skin reactions were more frequent in the LT group than in the MIPS group (18.2% vs. 8.7%), but the difference was not statistically significant (*p* = 0.249). A single implant loss occurred in the LT group 18 months after surgery and was unrelated to failed osseointegration or the surgical technique, being caused by recurrent cholesteatoma. **Conclusions**: MIPS was associated with a significantly shorter operative time than the LT. Both MIPS and LT appear to be safe techniques. No statistically significant differences were detected between the groups with respect to adverse skin reactions or implant survival during the follow-up period.

## 1. Introduction

Bone-anchored hearing devices (BAHDs) are indicated in patients with conductive or mixed hearing loss when reconstructive surgery or hearing aids are ineffective or cannot be applied. They bypass damaged or malformed parts of the external or middle ear and transmit sound directly to the inner ear via the bone. They are also used in patients with single-sided deafness (SSD) to transmit sound to the contralateral normal-hearing ear [1,2]. Currently, there is a variety of BAHD solutions on the market, which can be divided into passive percutaneous, passive transcutaneous, and active.

Percutaneous BAHDs consist of a titanium implant placed into the temporal bone, an abutment protruding through the skin, and a sound processor, which is attached to the abutment. The sound processor receives sounds and converts them into vibrations, which are then transmitted through the abutment, implant, and bone to the inner ear, thereby enabling hearing [1,3].

BAHDs were invented in the 1970s and have evolved over the past four decades [3]. In addition to advances in device technology, surgical implantation techniques have also evolved considerably. For percutaneous systems, the dermatome technique and linear incision technique (LT) with soft tissue reduction were the gold standard methods for many years. However, the dermatome technique was found to increase the risk of infections and necrosis of the skin surrounding the abutment [4]. The LT with soft tissue preservation, implemented in 2010, resulted in improved skin sensation and better cosmetic outcomes. Over the following years, both the scientific literature and presentations and discussions at scientific meetings increasingly emphasized the importance of abandoning soft tissue reduction as a means of reducing the rate of complications [4,5]. The Minimally Invasive Ponto Surgery (MIPS) technique was developed in 2015. It is a suture-free procedure that allows placement of a titanium implant directly into the temporal bone. Drilling a hole in the bone is done through a special cannula using specially designed drills. Reduced soft tissue trauma promotes faster healing and shorter operating time [6]. In 2022, Oticon Medical AB (Askim, Sweden) simplified the drilling process by combining two drills into one, creating the MONO kit.

Although several studies have compared LT and MIPS, most have been limited by relatively short follow-up periods, different implant systems used (Cochlear Ltd. (Sydney, Australia)/Oticon Medical AB), or small sample sizes. The present study aimed to compare perioperative and postoperative clinical outcomes of the MIPS and LT methods in patients undergoing BAHD implantation using the same Ponto^®^ implant system. It was hypothesized that MIPS would be associated with shorter operative time and a lower rate of postoperative adverse events without compromising implant survival.

## 2. Materials and Methods

### 2.1. Study Design

This study was a non-randomized, retrospective review of two series of patients who underwent Ponto^®^ (Oticon Medical AB) implantation at the Department of Otolaryngology and Laryngological Oncology of Poznan University of Medical Sciences in 2012–2014 and 2020–2022. All participants received a 4 mm long titanium Ponto Implant (Oticon Medical AB). The analysis focused on the course of surgery (type of anesthesia, median operative time measured from the initiation of local anesthesia to the placement of the healing cap, selected abutment length, intraoperative difficulties, and complications), healing process (adverse skin reactions according to the Holgers score system, which was used to assess skin reactions during the first 3 postoperative months), and long-term conditions of the implant site (the need for revision surgery, implant loss rate). Data were obtained from patients’ medical records and telephone interviews. To ensure a comparable follow-up period between the two groups, follow-up was censored at 48 months. For patients with a longer follow-up, only data collected within the first 48 months after implantation were included in the analysis. The median follow-up was 31 months (range: 4–48 months) in the LT group and 34 months (range: 3–48 months) in the MIPS group. The last patient was implanted in March 2022, and follow-up was completed in April 2026. The study protocol was reviewed and approved by the regional ethics committee (decision number: 655/22).

### 2.2. Patients

Surgical and follow-up data were collected from 101 patients. The LT was performed in 55 patients between 2012 and 2014 (LT group). Among them, 28 underwent procedures involving soft tissue reduction, while 27 were treated using the tissue preservation technique. Minimally invasive procedures were performed in 46 individuals between 2020 and 2022 (MIPS group). The study group included patients aged 15–82 years, with the predominance of individuals within the 51–75-year age group. The median age at implantation was 57 years and was significantly higher in the MIPS group than in the LT group (61 vs. 55 years; *p* = 0.003). There was no significant sex predominance. Chronic otitis media was the most frequent indication in both the MIPS (69.6%) and LT (63,6%) groups. It was followed by otosclerosis (13 cases, 12.9%) and single-sided deafness (6 cases, 5.9%). Most operated patients had comorbidities. The most common was hypertension, followed by hypothyroidism. Other comorbidities were less common and included diabetes mellitus, coronary artery disease, and a history of prior oncological treatment. Patients’ characteristics are presented in Table 1.

### 2.3. Surgery

Most surgeries (except two) were performed under local anesthesia. Only two patients underwent surgery under general anesthesia, as they were minors. Skin thickness was measured with a needle at the surgical site before local anesthetic injection to choose the appropriate abutment length. For perioperative prophylaxis, patients were administered three doses of cefuroxime at 8 h intervals.

**Figure 1:**
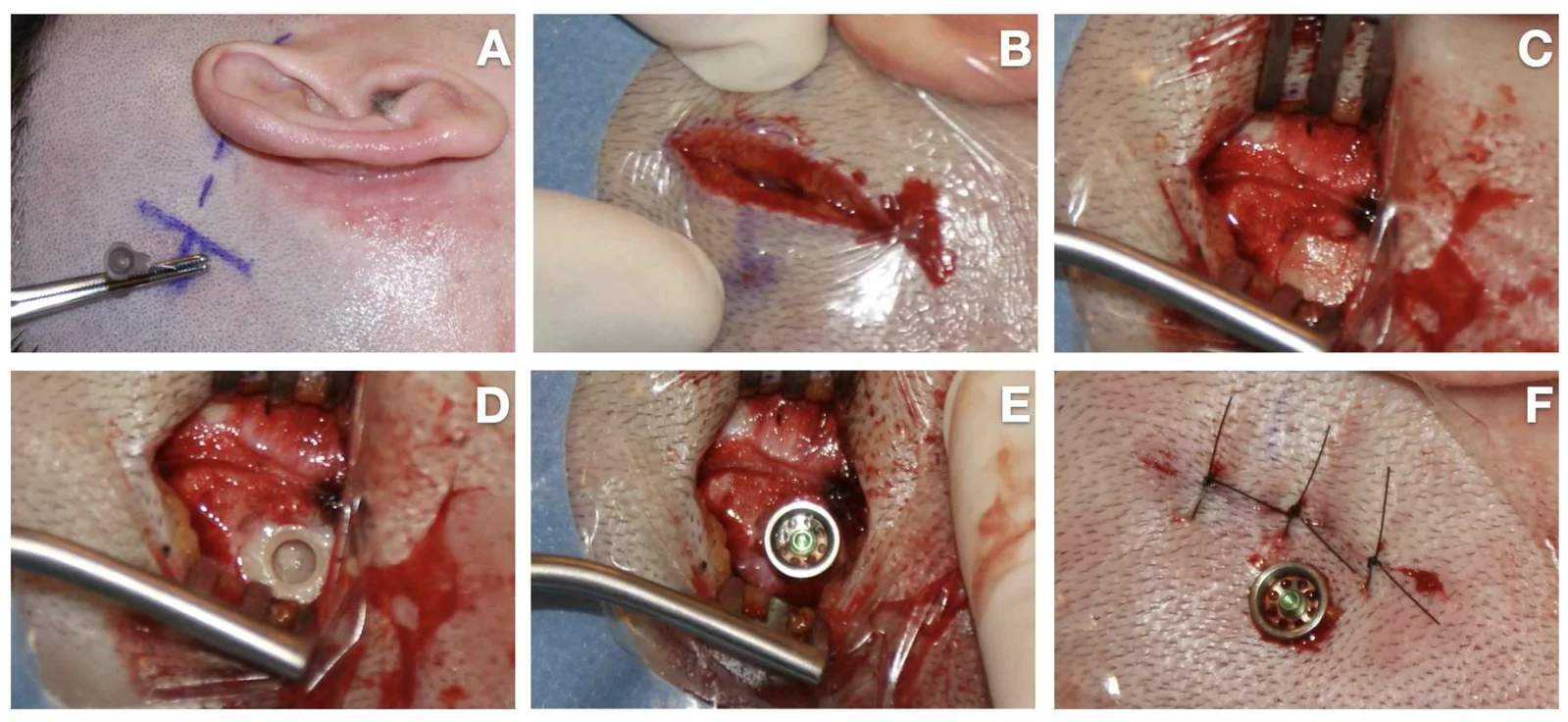
Ponto^®^ bone conduction implant insertion using linear incision technique (right site): (**A**) marked surgical area for implant and abutment placement, (**B**) performed skin and subcutaneous tissue incision, (**C**) removed periosteum from bone surface before drilling, (**D**) prepared hole for implant insertion, (**E**) inserted implant with abutment, (**F**) skin sutures and visible abutment protruding through the skin.

Using the LT, a 20–40 mm long vertical incision was made in the postauricular area, 50–55 mm from the external ear canal. A musculoperiosteal flap was raised. The periosteum was removed from the future implant location. A hole in the temporal bone was made using two drills: a guided drill and a widening drill, followed by placement of the implant with the abutment. During drilling and implantation, continuous aqua cooling was applied. The skin above the abutment was removed using a biopsy punch. In 2012, because only 6 mm abutments were available, soft tissue reduction, including the removal of muscle or subcutaneous tissue, was performed in the majority of patients. In the second half of 2013, following the availability of 9 mm and 12 mm abutments, the tissue preservation technique was gradually introduced. Subcutaneous and skin sutures were placed, and a healing cap with an ointment dressing was applied (Figure 1).

**Figure 2:**
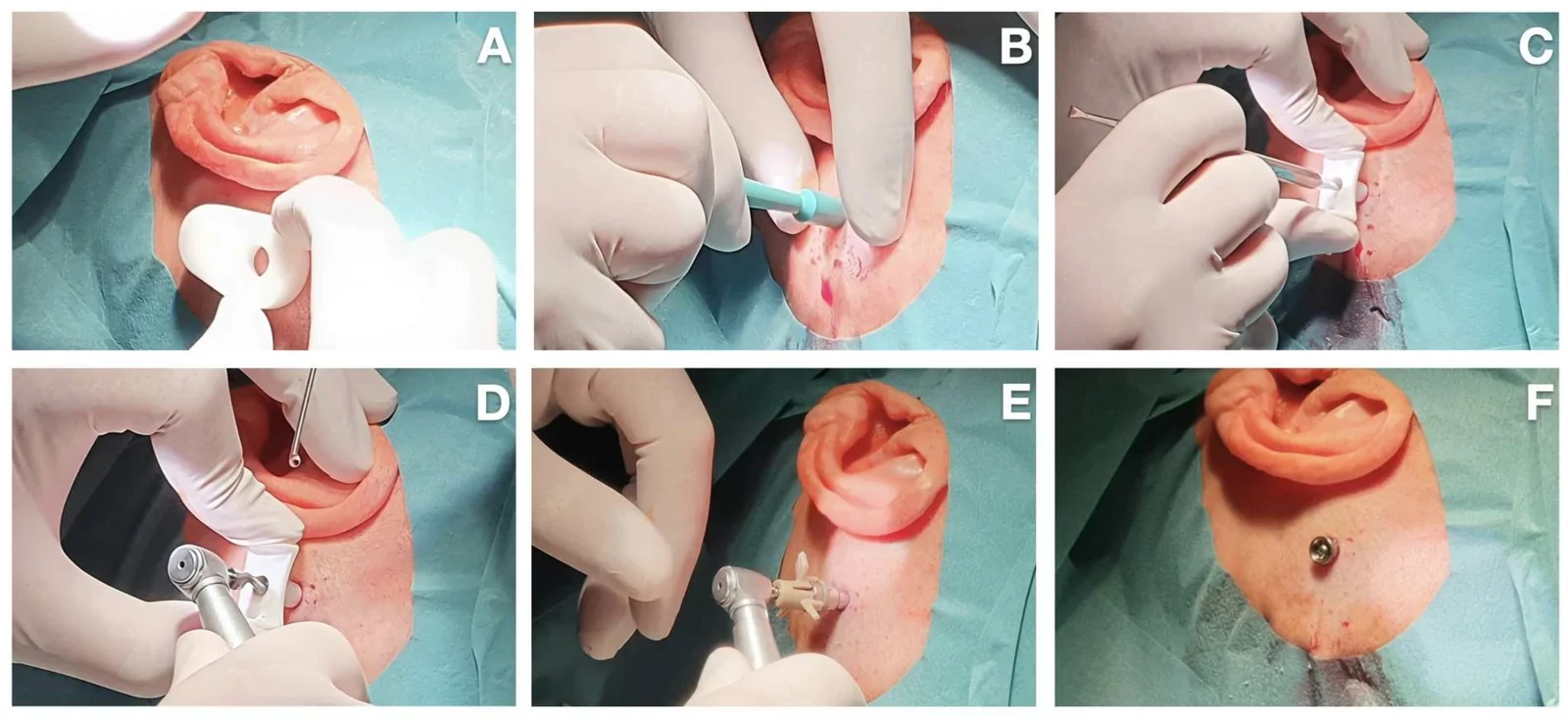
Minimally Invasive Ponto^®^ Surgery (left site): (**A**) choice of surgical area for abutment and processor placement, (**B**) skin incision using biopsy punch, (**C**) periosteum removal, (**D**) drilling into bone through the cannula and preparation of a hole for implant, (**E**) implant insertion, (**F**) implant inserted into bone and visible abutment protruding through the skin.

In the minimally invasive technique, a 5 mm hole was made using a biopsy punch. After creating an opening in the skin and subcutaneous tissue, the periosteum was removed from the surgical field. A hole was drilled in the bone through a special cannula using specially designed drills. In the two-drill technique, a guided drill and a widening drill were used, and in the MONO procedure (performed in 3 patients), a single drill was used. After creating a hole in the bone, the implant with the abutment was inserted. No sutures were required with either minimally invasive method. A healing cap with an ointment dressing was applied (Figure 2).

All patients were discharged from the hospital one day after surgery. Routine postoperative antibiotics were not prescribed. The first follow-up visit was scheduled for 10 days postoperatively to assess healing and remove sutures when necessary. Subsequent follow-up visits were planned approximately 4–6 weeks later to evaluate the surgical site and fit the sound processor.

### 2.4. Statistical Analysis

Statistical analysis was performed using Fisher’s exact test for categorical variables and the Mann–Whitney U test for continuous variables due to the small number of events. Time of surgery was compared between groups using the Mann–Whitney U test. For dichotomous outcomes, odds ratios (ORs) with 95% confidence intervals (CIs) were calculated. Revision-free survival was estimated using the Kaplan–Meier method, and survival curves for the LT and MIPS groups were compared using the two-sided log-rank test. Multivariable Firth-penalized logistic regression was used to perform multivariable analysis. Regression results were reported as adjusted odds ratios (aORs) with profile-likelihood 95% confidence intervals and *p*-values. Regression analyses were based on complete cases. The results were considered statistically significant at *p* < 0.05.

## 3. Results

### 3.1. Surgery

The median time of the surgery was 40 min (range: 20–70 min) in the LT group and 30 min (range: 15–75 min) in the MIPS group, and the difference was statistically significant (*p* < 0.001). The longest procedure in the MIPS group (75 min) occurred in a patient who required conversion to the linear incision technique.

The chosen abutment lengths differed between the groups: the most common length in the LT group was 6 mm, while in the MIPS group, it was 9 mm, followed by 12 mm. The differences were statistically significant (*p* < 0.001, *p* = 0.022, *p* < 0.001, respectively). In the LT group, the choice of abutment length was determined primarily by the availability of different abutment lengths at the time of surgery rather than by clinical considerations.

The most common intraoperative difficulty in both groups was bleeding (three cases in the LT group and one patient in the MIPS group). In one LT patient, dural exposure without damage or cerebrospinal fluid leakage occurred; another patient showed exposure of the sigmoid sinus without injury to its wall. In one MIPS patient, conversion to the LT was required due to the inability to achieve proper implant placement and stability because of an irregular temporal bone surface.

Surgical results are presented in Table 2.

### 3.2. Healing Process and Long-Term Conditions of the Implant Site

Overall, 20 patients (19.8%) had postoperative local complications.

In the LT group, mild soft tissue reactions (Holgers grades 1 and 2) were observed in 10 cases (18.2%) and managed conservatively. Three patients (5.5%) developed soft tissue overgrowth around the abutment (without signs of infection). They required revision surgeries, which were performed 1, 4, and 18 months after the implantation, respectively. One patient had implant loss (18 months after the surgery) due to extensive cholesteatoma recurrence. None of the patients required explantation.

In the MIPS group, mild soft tissue complications (Holgers 1 and 2) were noted in four patients (8.7%), and all were managed conservatively. Two patients in the MIPS group (4.3%) required an in-office revision surgery (excision of the overgrowing skin around the abutment) (25 and 47 months after surgery). There was no implant loss and none of the patients required explantation.

**Figure 3:**
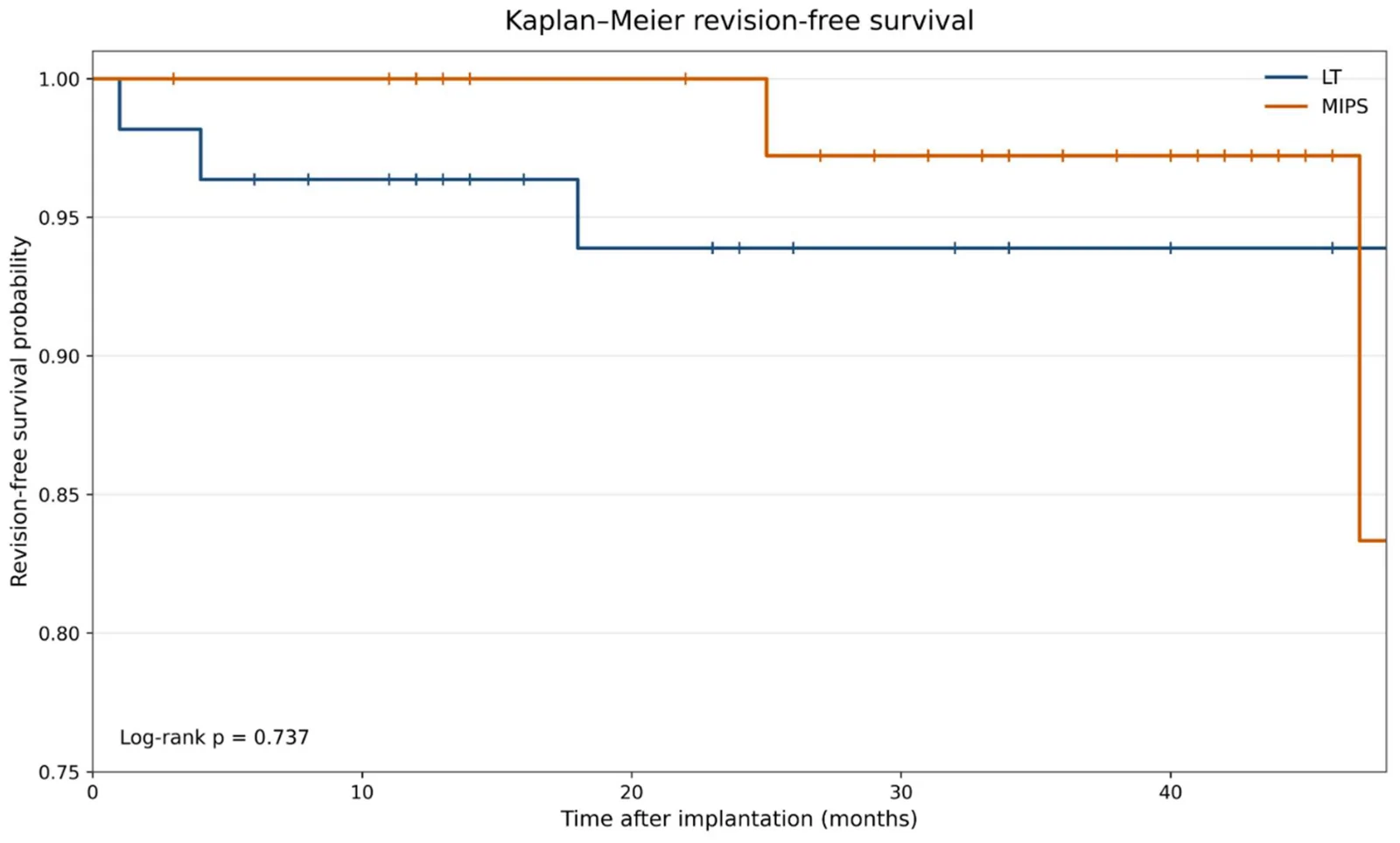
Kaplan–Meier curves for revision-free survival in the LT and MIPS groups during the 48-month follow-up period.

The incidence of adverse skin reactions (Holgers grades 1–2) was almost twofold lower in the MIPS group than in the LT group. Although this difference did not reach statistical significance (*p* = 0.249), it may still be clinically meaningful. The relatively small sample size may have limited the statistical power of the analysis. Within the LT cohort, no differences in the incidence of complications were observed between patients undergoing soft tissue reduction and those managed with the tissue preservation approach (17.9% vs. 18.5%). Moreover, no statistically significant differences were found between the LT and MIPS groups regarding revision surgery or implant loss (*p* = 1.000). During the follow-up period, revision surgery was needed in 3 of 55 patients (5.5%) in the LT group and 2 of 46 patients (4.3%) in the MIPS group. The estimated 48-month revision-free survival was 93.9% in the LT group and 83.3% in the MIPS group. Kaplan–Meier curves did not differ significantly between the groups (log-rank χ^2^ = 0.113, df = 1, *p* = 0.737). Median revision-free survival was not reached in either group. Because only five revisions occurred and relatively few patients remained under follow-up at 48 months, the survival estimates at the end of the observation period should be interpreted with caution (Figure 3). Postoperative complications are presented in Table 3, while a summary of clinical outcomes is shown in Table 4. Multivariate analysis showed no significant association between adverse skin reactions and age, comorbidities, operative time, abutment length, and surgical technique (LT with soft tissue reduction, LT with soft tissue preservation, MIPS) (Table 5).

## 4. Discussion

Currently, BAHD implantations are mostly performed in patients with congenital malformations of the external or middle ear, chronic otitis media, ossicular pathology, and other cases in which surgical methods of sound transmission system reconstruction and hearing aids are ineffective or contraindicated. Another group that can benefit from BAHD is individuals with single-sided deafness [3]. Despite enormous technological progress and the increasing popularity of so-called active implant systems, which provide very good hearing and aesthetic results, percutaneous implants remain the optimal solution for certain groups of patients [7]. An important advantage of passive percutaneous BAHD with the titanium implant and abutment is the fact that they produce much fewer artifacts during MRI imaging than systems with implanted magnets (passive transcutaneous and active), which makes them suitable for patients who require regular MRI testing of the head [8]. It refers to patients with chronic otitis media after canal wall-up cholesteatoma surgery, patients after acoustic neuroma surgery or radiotherapy, and individuals with some neurological diseases. Another significant advantage of percutaneous systems is the ability to perform implantation under local anesthesia, which is particularly important for older individuals and those with underlying health conditions that preclude the use of general anesthesia. Focusing on audiological indications, such systems offer the potential for use in cases of more profound hearing loss (bone conduction levels up to 65 dB HL). In our department, the choice of Ponto^®^ in the LT group was mostly connected with system availability at that time (implantation of magnetic systems in Poznan began in 2014), but in the MIPS group, the system was chosen especially in cases after cholesteatoma surgery requiring MRI monitoring and for elderly patients.

Intraoperative complications associated with all BAHD implantation techniques are generally rare (ranging from 0% to 13.3% of patients depending on the authors, implantation technique, and type of adverse event). They include drilling into a vein, dural exposure or rupture, bleeding from the skin, and drilling into an air cell [1,3,9,10]. When punch-only techniques were introduced, there were concerns that the more limited surgical view might increase the risk of bleeding or dural injury. However, available studies do not confirm higher intraoperative risk for minimally invasive techniques. De Stefano et al. [9] reported no intraoperative complications in either the MIPS or the LT groups in their cohort of 48 implanted patients. Some authors have reported a lower number of intraoperative complications with MIPS compared with the LT approach (9.1% vs. 13.3%; 1.8 vs. 4.9%), which is consistent with our clinical experience [1,10].

In recent years, punch-only techniques have been further developed in response to the growing interest in less invasive surgery and its potential clinical benefits. New surgical sets, including the MONO kit, have been introduced to shorten operative time. Ganeyev et al. [11] found the MONO system for BAHD implantation safer and more efficient for creating the hole in the bone for the implant in terms of energy efficiency and temperature control than the classic MIPS drill system (drill force and torque were measured during drilling in cow tibia at different feed rates). However, as this technique is relatively new, long-term outcomes have not yet been reported in the literature [10]. In our study group, the MONO procedure was used in three patients. Due to the small number of cases, it was not analyzed separately.

Published studies suggest that minimally invasive techniques may provide improved esthetic outcomes due to the absence of additional scalp scarring and reduced postoperative oedema, particularly in the early postoperative period. Shorter surgical time supports the use of the MIPS approach, as it is less stressful for patients and suitable for procedures performed under local anesthesia [1,9,10,12]. In our group, the MIPS procedure time was significantly shorter than in the cases of LT, which is consistent with the published data. In addition to its clinical advantages, shorter surgical time may also mean economic benefits. Overall procedural costs are lower due to, e.g., reduced operating room occupancy. Sardiwalla et al. [13] compared the minimally invasive punch technique with traditional surgical approaches for percutaneous BAHD implantation and reported a mean cost reduction of CAD 456.83 per procedure. Strijbos et al. [14] compared the average cost of MIPS and LT with tissue preservation and found that after 22 months of follow-up, MIPS was associated with a lower mean cost, with a difference of EUR 77.83 per patient.

The most common postoperative adverse events following BAHD surgery include skin irritation or granulation around the abutment, skin sagging, pain around the implant, loss of skin sensation, headache, and soft tissue overgrowth around the abutment [1]. Implant loss is much less common than skin adverse events, but may occur spontaneously or after trauma, especially in children, with reported rates ranging from 7.9% to 9.5% [1,14]. Calon et al. reported four cases of implant loss in the MIPS group (out of 33 patients) and one case in the LT group (out of 30 patients) [1]. Teunissen et al. [15] compared the 3-year outcomes of modified MIPS (m-MIPS; three-stage drilling: after creating a guide hole using the cannula guide drill with spacer, deepening the hole for a 4 mm implant using the same drill without spacer, then widening the hole with the cannula widening drill) with the original MIPS and the LT with soft tissue preservation. They showed that in m-MIPS, long-term implant survival was similar to that observed for the other techniques, and it was associated with fewer adverse skin reactions and postoperative complications over the 3-year follow-up period. Calon et al. [1] did not observe a significant difference in the rate of inflammation (Holgers ≥ 2) between the MIPS and LT groups; however, MIPS resulted in better esthetic results (less skin sagging, lower rates of wound dehiscence). At the same time, this group had a higher extrusion rate (12.1% in MIPS vs. 3.3% in LT), although the difference was not statistically significant (*p* = 0.19). During a 1-year follow-up, Di Giustino et al. [16] reported adverse events in 10.63% of cases in the LT group with subcutaneous tissue reduction, 3.12% in patients who underwent LT with tissue preservation, and 2.5% in the MIPS group; nevertheless, these differences were not statistically significant. Our observations regarding local inflammatory complications are similar to those reported by previously cited groups. No implant losses related to osseointegration or surgical technique were noted. Although 50.9% of patients in the LT group underwent soft tissue reduction, no significant increase in the incidence of adverse skin reactions was observed compared with those managed using the LT with tissue preservation approach.

### Strengths and Limitations

The strengths of our study are the large sample size; the use of the same implant type between the groups, which eliminated differences in the osseointegration process and skin reactions related to implant and abutment materials; and the same surgical team performing all procedures and follow-up visits.

A major limitation of this study is its retrospective comparison of two cohorts treated during different time periods. Consequently, the groups differed not only in surgical technique but also in calendar period, patient characteristics (age), and surgeon experience. These factors represent potential confounders and may have contributed to the observed differences independently of the surgical technique. Therefore, the results should be interpreted as descriptive and not as evidence of causal superiority of any one surgical technique.

## 5. Conclusions

MIPS was associated with a significantly shorter operative time than the LT. Both MIPS and LT appear to be safe techniques. No statistically significant differences were detected between the groups with respect to adverse skin reactions or implant survival during the follow-up period.

## Figures and Tables

**Table 1:** Patients’ characteristics.

	LT	MIPS	ALL	*p*-Value
**NUMBER**	55	46	101	
**AGE GROUP, *n* (%)**				
<18	2 (3.6)	0 (0)	2 (2.0)	0.499
18–50	20 (36.4)	13 (28.3)	33 (32.7)	0.404
51–75	32 (58.2)	28 (60.8)	60 (59.4)	0.840
>75	1 (1.8)	5 (10.9)	6 (5.9)	0.089
Median age (range)	55 (15–78)	61 (32–85)	57 (15–85)	0.003
**GENDER, *n* (%)**				
Female	30 (54.5)	25 (54.3)	55 (54.5)	1.000
Male	25 (45.5)	21 (45.7)	46 (45.5)	
**COMORBIDITIES, *n* (%)**				
Not reported	11 (20.0)	11 (23.9)	22 (21.8)	1.000
Hypertension	10 (18.2)	17 (36.9)	27 (26.7)	0.104
Hypothyroidism	5 (9.1)	6 (13.0)	11 (10.9)	0.754
Other	18 (32.7)	8 (17.4)	26 (25.7)	-
No data	11 (20.0)	4 (8.7)	15 (14.9)	-
**INDICATION, *n* (%)**				
Chronic otitis media	35 (63.6)	32 (69.6)	67 (66.3)	0.672
Otosclerosis	6 (10.9)	7(15.2)	13 (12.9)	0.562
Single-sided deafness	5 (9.1)	1 (2.2)	6 (5.9)	0.216
Other	9 (16.4)	6 (13.0)	15 (14.9)	-

**Table 2:** Surgical results.

	LT	MIPS	ALL	*p*-Value
**TYPE OF ANESTHESIA, *n* (%)**				
General	2 (3.6)	0	2 (2.0)	0.499
Local	53 (96.4)	46 (100.0)	99 (98.0)	
**IMPLANTATION SITE, *n* (%)**				
Right	33 (60.0)	25 (54.3)	58 (57.4)	0.686
Left	22 (40.0)	21 (45.7)	43 (42.6)	
**OPERATIVE TIME (minutes)**				
Median (range)	40 (20–70)	30 (15–75)	35 (15–75)	<0.001
**ABUTMENT LENGTH, *n* (%)**				
6	33 (60.0)	7 (15.2)	40 (39.6)	<0.001
9	15 (27.3)	25 (54.3)	40 (39.6)	0.022
12	2 (3.6)	14 (30.4)	16 (15.8)	<0.001
No data	5 (9.1)	0 (0)	5 (5.0)	-

**Table 3:** Postoperative complications.

	LT		MIPS	ALL	*p*-Value
	With Soft Tissue Reduction	With Soft Tissue Preservation			
**HOLGERS SCALE, *n* (%)**					
0 normal skin around the abutment	23 (82.1)	22 (81.5)	42 (91.3)	87 (86.1)	0.379
1 slight redness around the abutment	2 (7.1)	3 (11.1)	2 (4.3)	7 (6.9)	
2 redness, moistness, and moderate swelling	3 (10.8)	2 (7.4)	2 (4.3)	7 (6.9)	
3 redness, moistness, and swelling with tissue granulation requiring revision surgery	0 (0)	0 (0)	0 (0)	0 (0)	
4 signs of infection resulting in removal of the implant	0 (0)	0 (0)	0 (0)	0 (0)	
**REVISION SURGERY FOR TISSUE REDUCTION, *n* (%)**	1 (3.6)	2 (7.4)	2 (4.3)	5 (4.9)	0.722
**IMPLANT LOSS, *n* (%)**	0 (0)	1 (3.7)	0 (0)	1 (1)	0.267

*p*-values were calculated for Holgers grade 0 versus Holgers grades ≥ 1.

**Table 4:** Summary of clinical outcomes—univariate analysis.

	LT	MIPS	OR (95% CI)	*p*-Value
**OPERATIVE TIME** [min; median (range)]	40 (20–70)	30 (15–75)	N/A	<0.001
**ADVERSE SKIN REACTIONS** [%]	18.2	8.7	2.333 (0.679–8.010)	0.249
**REVISION SURGERY** [%]	5.5	4.3	1.269 (0.203–7.942)	1.000
**IMPLANT LOSS** [%]	1.8	0	2.559 (0.102–64.351) *	1.000

* 0.5 continuity correction of 0.5 applied because no implant loss occurred in the MIPS group.

**Table 5:** Multivariate analysis of factors associated with adverse skin reactions (age, comorbidities, operative time, abutment length, and surgical technique (LT with soft tissue reduction, LT with soft tissue preservation, MIPS)).

	aOR (95% CI)	*p*-Value
**AGE**	0.965 (0.927–1.001)	0.058
COMORBIDITIES *	3.424 (0.674–27.404)	0.147
**OPERATIVE TIME**	1.024 (0.963–1.083)	0.414
**ABUTMENT LENGTH**	1.104 (0.746–1.648)	0.614
SURGICAL TECHNIQUE **		
• **LT vs. MIPS**	2.496 (0.523–14.168)	0.253
• **LT with soft tissue reduction vs. MIPS**	5.391 (0.729–51.950)	0.100
• **LT with soft tissue preservation vs. MIPS**	1.976 (0.354–11.924)	0.431
• **LT with soft tissue reduction vs. LT with soft tissue preservation**	1.751 (0.324–11.456)	0.519

* Comorbidities were dichotomized as present versus absent. ** Pairwise comparisons between surgical techniques were obtained from the same regression model using MIPS as the reference category.

## Data Availability

The original contributions presented in this study are included in the article. Further inquiries can be directed to the corresponding author.

## References

[1] Calon T.G.A., Johansson M.L., de Bruijn A.J.G., van den Berge H., Wagenaar M., Eichhorn E., Janssen M.M.L., Hof J.R., Brunings J.W., Joore M.A. (2018). Minimally Invasive Ponto Surgery Versus the Linear Incision Technique With Soft Tissue Preservation for Bone Conduction Hearing Implants: A Multicenter Randomized Controlled Trial. Otol. Neurotol..

[2] Crowson M.G., Tucci D.L. (2016). Mini Review of the Cost-Effectiveness of Unilateral Osseointegrated Implants in Adults: Possibly Cost-Effective for the Correct Indication. Audiol. Neurootol..

[3] Lagerkvist H., Carvalho K., Holmberg M., Petersson U., Cremers C., Hultcrantz M. (2020). Ten Years of Experience with the Ponto Bone-Anchored Hearing System: A Systematic Literature Review. Clin. Otolaryngol..

[4] Hultcrantz M. (2011). Outcome of the Bone-Anchored Hearing Aid Procedure without Skin Thinning: A Prospective Clinical Trial. Otol. Neurotol..

[5] Van de Berg R., Stokroos R.J., Hof J.R., Chenault M.N. (2010). Bone-Anchored Hearing Aid: A Comparison of Surgical Techniques. Otol. Neurotol..

[6] Caspers C.J.I., Kruyt I.J., Mylanus E.A.M., Hol M.K.S. (2021). A Clinical Evaluation of Minimally Invasive Ponto Surgery with a Modified Drill System for Inserting Bone-Anchored Hearing Implants. Otol. Neurotol..

[7] Arndt S., Cantore I., Smeds H., Goldberg-Bockhorn E., Lok W., Marco J., Röösli C., Gawęcki W. (2024). Expert opinion on candidacy for bone conduction hearing implants Osia System and Baha Connect System. Otolaryngol. Pol..

[8] Ellsperman S.E., Nairn E.M., Stucken E.Z. (2021). Review of Bone Conduction Hearing Devices. Audiol. Res..

[9] De Stefano S., Mochi P., Murri A., Cuda D. (2021). Comparison between Linear Incision and Punch Techniques for Bone Anchored Hearing Aid Surgery. Acta Otorhinolaryngol. Ital..

[10] Cruz L.D.S., Danieli F., Håkansson M.Å., Johansson M.L., dos Santos F.R., Mirândola Barbosa Reis A.C., Hyppolito M.A. (2023). Minimally Invasive Surgery as a New Clinical Standard for Bone Anchored Hearing Implants—Real-World Data from 10 Years of Follow-Up and 228 Surgeries. Front. Surg..

[11] Ganeyev M., Shah F.A., Thomsen P., Palmquist A., Johansson M.L. (2025). Mechanical and Thermal Efficiency of a Single Drill System for Bone-Anchored Hearing Implants. PLoS ONE.

[12] Caspers C.J.I., Kruyt I.J., Mylanus E.A.M., Hol M.K.S. (2020). Six-Month Clinical Outcomes for Bone-Anchored Hearing Implants: Comparison Between Minimally Invasive Ponto Surgery and the Linear Incision Technique with Tissue Preservation. Otol. Neurotol..

[13] Sardiwalla Y., Jufas N., Morris D.P. (2017). Direct Cost Comparison of Minimally Invasive Punch Technique Versus Traditional Approaches for Percutaneous Bone Anchored Hearing Devices. J. Otolaryngol. Head. Neck Surg..

[14] Strijbos R.M., Straatman L.V., Calon T.G.A., Johansson M.L., de Bruijn A.J.G., van den Berge H., Wagenaar M., Eichhorn E., Janssen M., Jonhede S. (2021). Long-Term Outcomes of the Minimally Invasive Ponto Surgery vs. Linear Incision Technique with Soft Tissue Preservation for Installation of Percutaneous Bone Conduction Devices. Front. Neurol..

[15] Teunissen E., Caspers C., Kruyt I., Mylanus E., Hol M. (2025). Long-Term Clinical Outcomes for Bone-Anchored Hearing Implants: 3-Year Comparison Between Minimally Invasive Ponto Surgery and the Linear Incision Technique with Tissue Preservation. Otol. Neurotol..

[16] Di Giustino F., Vannucchi P., Pecci R., Mengucci A., Santimone R., Giannoni B. (2018). Bone-Anchored Hearing Implant Surgery: Our Experience with Linear Incision and Punch Techniques. Acta Otorhinolaryngol. Ital..

